# Metabolic Engineering of *Escherichia coli* for Production of Mixed-Acid Fermentation End Products

**DOI:** 10.3389/fbioe.2014.00016

**Published:** 2014-05-23

**Authors:** Andreas H. Förster, Johannes Gescher

**Affiliations:** ^1^Institute of Applied Biosciences, Karlsruhe Institute of Technology, Karlsruhe, Germany

**Keywords:** *E. coli*, mixed-acid fermentation, carbon source, succinate, lactate, ethanol, acetate

## Abstract

Mixed-acid fermentation end products have numerous applications in biotechnology. This is probably the main driving force for the development of multiple strains that are supposed to produce individual end products with high yields. The process of engineering *Escherichia coli* strains for applied production of ethanol, lactate, succinate, or acetate was initiated several decades ago and is still ongoing. This review follows the path of strain development from the general characteristics of aerobic versus anaerobic metabolism over the regulatory machinery that enables the different metabolic routes. Thereafter, major improvements for broadening the substrate spectrum of *E. coli* toward cheap carbon sources like molasses or lignocellulose are highlighted before major routes of strain development for the production of ethanol, acetate, lactate, and succinate are presented.

## Introduction

Over decades, *Escherichia coli* has been studied regarding the multitude of factors that determine its physiology and the different phenotypes it can adopt. The molecular toolbox, enabling genetic engineering and studying of regulation and gene expression, is presumably the largest that exists for one particular organism (Richter and Gescher, 2012). Hence, it is not surprising that *E. coli* has been widely used by applied microbiologists to try to steer the metabolism of this organism toward the production of molecules with biotechnological value.

This topic gains increasing interest due to the development of our society toward a bioeconomy. Chemical production routines using fossil fuels as substrates are more and more being replaced by sustainable processes that work with biological catalysis and renewable substrates. Interestingly, strains developed several years ago are still the starting points for the development of new strains that either broaden the spectrum of producible substances or increase the efficiency of an applied process.

With this in mind, we review studies from several decades to show the strain development for production of mixed-acid fermentation substances, with a focus on anaerobic processes. After an overview of metabolic capabilities and regulatory mechanisms, developments regarding the expansion of usable substrate spectrum will be described. Thereafter, strategies for the production of ethanol, acetate, lactate, and succinate are presented.

## Central Metabolism

*Escherichia coli* is a facultative anaerobic, Gram-negative organism and capable of using a wide spectrum of organic carbon sources for heterotrophic growth. The availability of electron acceptors triggers the strategies used for energy production – respiration or fermentation. In most studies that describe the use of *E. coli* for applied processes, sugars are used as carbon and electron source. Their import can be achieved via different uptake machineries. Glucose, for example, is mainly imported and simultaneously phosphorylated to glucose-6-phosphate using the phosphotransferase system (Hunter and Kornberg, 1979; Escalante et al., 2012). Nevertheless, it has been shown that the galactose and mannose uptake machineries can also translocate glucose, which then enters the cytoplasm in its unphosphorylated form and is converted to glucose-6-phosphate by glucokinase (Steinsiek and Bettenbrock, 2012). The glycolysis pathway processes the phosphorylated sugar into two molecules of pyruvate, which is accompanied by the release of two ATP and two NADH molecules. Under oxic conditions, pyruvate is converted to acetyl-CoA and carbon dioxide by the pyruvate dehydrogenase complex. This multimeric enzyme is downregulated under anaerobic conditions and is controlled by the NADH/NAD^+^ ratio (de Graef et al., 1999) as well as the pyruvate concentration (Quail et al., 1994). Under oxic conditions, acetyl-CoA is further processed within the citric acid cycle. This gives rise to the production of more ATP and reducing equivalents.

*Escherichia coli* is also able to respire under anoxic conditions, and can use a variety of substances in the absence of oxygen as electron acceptors (Ingledew and Poole, 1984; Stewart, 1993). Nevertheless, the absence of oxygen triggers a downregulation of the citric acid cycle, which leads to an incomplete oxidation of sugars. Under these conditions, acetate is formed as the main product. Pyruvate dehydrogenase is replaced by pyruvate formate lyase, which prevents the release of NADH during pyruvate consumption and instead catalyzes the formation of formate and acetyl-CoA. The latter is converted to the accumulating acetate by the activity of phosphotransacetylase (*pta*) and acetate kinase (*ack*) (Trotter et al., 2011). The two central regulators fumarate-nitrate-reduction (FNR) and ArcAB (anoxic redox control) mediate the distinction between oxic and anoxic metabolism (Sawers and Suppmann, 1992).

**Figure 1:**
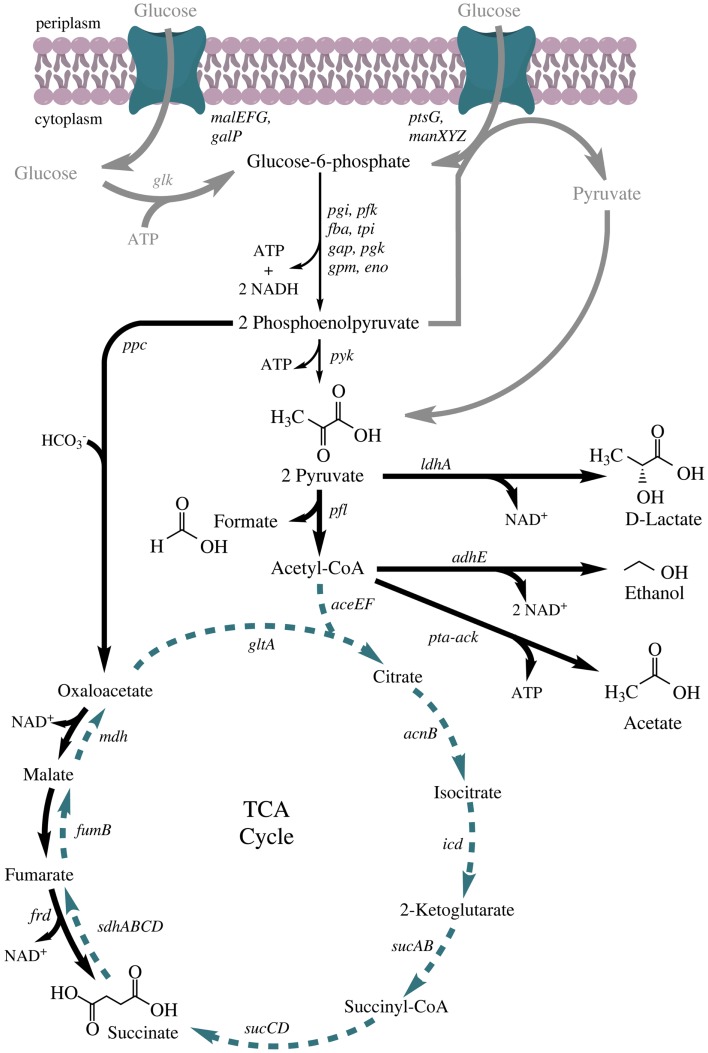
**Anaerobic fermentative metabolism in *Escherichia coli***. Chemical structures are shown for all mixed-acid fermentation products and pyruvic acid. Bold gray arrows: glucose transport systems; thin black arrows: glycolysis; bold black arrows: fermentative reactions; dashed, green arrows: TCA cycle, only anabolic functions, completely active under oxic conditions. Genes: *malEFG* (maltose ABC transporter), *galP* (galactose:H^+^ symporter), *ptsG* (fused glucose-specific PTS enzyme: IIB and IIC component), *manXYZ* (mannose PTS permease), *glk* (glucokinase), *pgi* (glucose-6-phosphate isomerase), *pfk* (6-phosphofructokinase), *fba* (fructose-bisphosphate aldolase), *tpi* (triosephosphate isomerase), *gap* (glyceraldehyde 3-phosphate dehydrogenase), *pgk* (phosphoglycerate kinase), *gpm* (phosphoglycerate mutase), *eno* (enolase), *pyk* (pyruvate kinase), *ppc* (phosphoenolpyruvate carboxylase), *ldhA* (lactate dehydrogenase), *pfl* (pyruvate formate lyase), *aceEF* (pyruvate dehydrogenase complex), *adhE* (alcohol dehydrogenase), *pta* (phosphate acetyltransferase), *ack* (acetate kinase), *gltA* (citrate synthase), *acnB* (aconitase), *icd* (isocitrate dehydrogenase), *sucA* (2-oxoglutarate decarboxylase), *sucB* (2-oxoglutarate dehydrogenase), *sucCD* (succinyl-CoA synthetase), *sdhABCD* (succinate dehydrogenase), *fumB* (fumarate hydratase), *frd* (fumarate reductase), and *mdh* (malate dehydrogenase).

Under fermentative conditions, a mixture of succinate, formate, acetate, lactate, and ethanol is produced to maintain redox balance (Clark, 1989). Ethanol formation is established using alcohol dehydrogenase (*adhE*), which catalyzes the reaction from acetyl-CoA to ethanol with the consumption of two NADH molecules. The production of lactate is catalyzed by the soluble lactate dehydrogenase (*ldhA*) via reduction of pyruvate (consumption of one NADH molecule). Succinate formation starts with the carboxylation of phosphoenolpyruvate to oxaloacetate by PEP-carboxylase (*ppc*), and is subsequently achieved via the activity of malate dehydrogenase (*mdh*), fumarase (*fumB*), and fumarate reductase (*frd*) (Figure 1).

## The Global Regulators ArcAB and FNR

ArcA and FNR greatly influence the expression of genes involved in central metabolism (Salmon et al., 2003, 2005; Constantinidou et al., 2006). In fact, they are the most important regulators involved in aerobic and anaerobic growth (Gunsalus and Park, 1994; Unden et al., 1995; Unden and Bongaerts, 1997).

**Figure 2:**
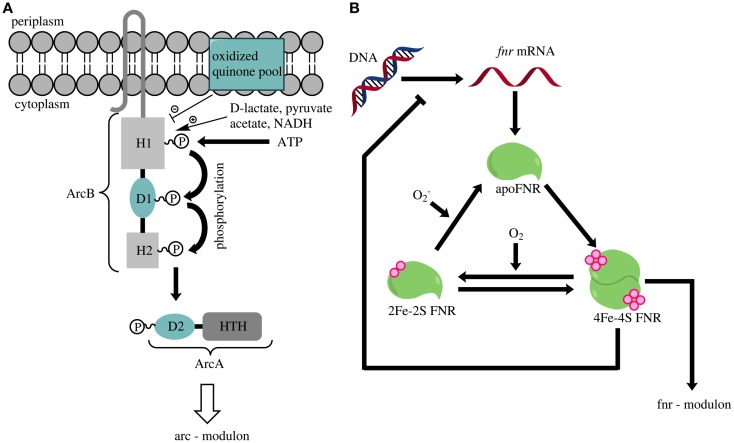
**(A)** Overview of the activation of the ArcAB two-component system [according to and modified from Liu et al. (2009)]. An accumulation of lactate, pyruvate or NADH triggers a phosphorylation cascade in ArcB that finally leads to the phosphorylation of ArcA. ArcA is depicted as a two-component protein containing the secondary receiver domain D2 and a helix-turn-helix domain (HTH). Oxidized quinone molecules negatively modulate the ArcB activity. **(B)** Schematic overview of FNR-regulator activation (according to and modified from Tolla and Savageau, 2010). Oxygen inactivates the active dimeric form of FNR that contains one 4Fe-4S-cluster per monomer (4Fe-4S FNR). Continuous production of new FNR molecules and reactivation of the inactive 2Fe-2S-form (2Fe-2S FNR) or the apoenzyme (apo FNR) leads to constant cycling of the three FNR-forms. The absence of oxygen triggers a rapid accumulation of the 4Fe-4S-form, which dimerizes and thereby becomes an active transcription factor.

The two-component ArcAB system consists of the membrane-associated sensor kinase ArcB and the cytoplasmic response regulator ArcA (Iuchi and Lin, 1988; Iuchi et al., 1990) (Figure 2A). ArcB has three cytoplasmic domains (H1, D1, and H2) and two transmembrane segments (Iuchi et al., 1990; Kwon et al., 2000). Phosphorylation of H1 is inhibited by oxidized quinones within the cytoplasmic membrane (Georgellis et al., 2001; Bekker et al., 2010) and stimulated by lactate, acetate, and pyruvate (Georgellis et al., 1999). Phosphorylation of H1 leads via several steps to formation of phosphorylated ArcA (Georgellis et al., 1997). This modified protein regulates the expression of numerous genes involved in energy metabolism. It represses genes contributing to respiration and activates those involved in fermentation (Malpica et al., 2006; Liu et al., 2009). For example, the TCA cycle, almost exclusively contributing to anabolic reactions under anoxic conditions, is upregulated in an *arcA* deletion strain during nitrate respiration (Prohl et al., 1998; Toya et al., 2012). Therefore, Pettinari et al. (2008) suggested that *arcA* deletion strains could be promising candidates for the production of reduced bioproducts like polyhydroxyalkanoates. The rationale behind this assumption is that the upregulation of citric acid cycle enzymes under anoxic conditions could potentially lead to elevated concentrations of NADH or NADPH. Beside the above described functions, the ArcAB system is also involved in aerobic hydrogen peroxide resistance (Loui et al., 2009) and microaerobic redox regulation (Alexeeva et al., 2003).

The FNR regulatory protein is the second global regulator for energy metabolism in *E. coli*. It is part of the Crp-Fnr superfamily (Korner et al., 2003) and contains a helix-turn-helix motif with a nucleotide-binding domain (Spiro and Guest, 1990). Expression of *fnr* is not coupled to growth conditions. Consequently, equal amounts of FNR are available in the cell under oxic and anoxic conditions (Unden and Duchene, 1987). Three different forms of FNR occur within the cell: (i) the apoenzyme (apoFNR), (ii) a monomeric FNR with a [2Fe-2S] cluster, and (iii) the homodimer containing one [4Fe-4S] cluster per monomer (Jervis et al., 2009; Tolla and Savageau, 2010). Of these three forms, only the homodimer functions as a transcriptional regulator (Shalel-Levanon et al., 2005). The availability of oxygen within the cell leads to monomerization of the homodimer and the loss of the Fe-S cluster (Figure 2B) (Lazazzera et al., 1996). The absence of oxygen causes a rapid increase of the homodimer concentration (Tolla and Savageau, 2010). FNR regulates gene expression on a transcriptional level. In summary, FNR activates the expression of genes involved in anaerobic fermentation and respiration, whereas it causes a downregulation of genes essential for aerobic respiration (Unden et al., 1995).

## Enabling the Consumption of Cost-Efficient and Abundant Carbon Sources

The availability of cost-efficient and abundant carbon sources is a requirement for biotechnological production of fermentation products by *E. coli*. These carbon compounds include lignocellulose, molasses, and glycerol (da Silva et al., 2009). Regarding the utilization of waste streams, the focus of research so far has been on ethanologenic strains of *E. coli*, most probably due to the growing demand for biofuels.

*Escherichia coli* is able to produce ethanol from pretreated wheat straw (Saha and Cotta, 2011; Saha et al., 2011) or marine algal hydrolyzates (Kim et al., 2011) without further heterologous gene expression. However, the accumulation of pretreatment side products, like furfural or phenolic compounds, inhibits microbial growth, and hampers fermentation (Mills et al., 2009). Consequently, Geddes et al. (2011) developed an ethanologenic *E. coli* strain with increased resistance to the toxic side products of hydrolysis. They used metabolic evolution experiments to isolate *E. coli* cells that are able to grow in a medium containing 60% phosphoric acid hydrolyzate – conditions toxic to the parent strain. The authors indicate that toxic side products can serve as a barrier to contamination with unwanted organisms, which could reduce fermentation costs. Wang et al. (2013) developed in a rational approach a furfural-resistant *E. coli* strain. They deleted *yqhD*, a gene encoding an alcohol dehydrogenase, and inserted the genes *fucO* (encoding an NADH-dependent furfural oxidoreductase) and *ucpA* (a cryptic gene encoded next to a sulfur assimilation operon) into the genome of an ethanologenic strain. The result was a reduction of furfural, and consequently furfural tolerance increased.

The ability for sucrose utilization is not prevalent among all cultivated *E. coli* strains. Only half of them are capable of using sucrose as a sole carbon source (Jahreis et al., 2002). Sucrose is the dominant carbohydrate in molasses, a side product of sugar production. Sucrose-utilizing *E. coli* strains can possess a plasmid-based (*scrKYAB* and *scrR*) or chromosomal system (*cscRAKB*). Shukla et al. (2004) expressed the *csc* genes in a sucrose-negative parent strain for the production of d-lactate from sucrose and molasses. They inserted the *csc* operon into the genome of an *E. coli* W3110 and determined lactate production in a sugar mixture and diluted molasses. As expected, *E. coli* could use diluted molasses as a sole carbon source. Hence, sucrose metabolism could be successfully transferred between different strains.

In contrast, wild type *E. coli* cells cannot thrive on cellobiose, a glucose dimer formed during cellulose hydrolysis. For cellobiose degradation, the β-glucosidase BglC from *Thermobifida fusca* was localized to the outer membrane of an ethanologenic *E. coli* strain. Transport to and attachment on the outer membrane was ensured by using a type V secretion system, the adhesin AIDA-I (Munoz-Gutierrez et al., 2012). AIDA-I is involved in the adhesion of enteropathogenic *E. coli* and had already been considered as a tool for surface display (Jose and Meyer, 2007). A plasmid-based expression system was constructed, and BglC was fused with a signal peptide and a translocation unit. Both signal sequences are essential for translocation, as well as protein attachment to the outer membrane. The resulting strain was able to ferment cellobiose to ethanol with a yield of 84% of the theoretical maximum (Munoz-Gutierrez et al., 2012).

Cellulose can be degraded by four different types of enzymes: endoglucanase, exoglucanase, β-glucosidase, and the recently discovered lytic polysaccharide monooxygenases (Lynd et al., 2002; Vaaje-Kolstad et al., 2010; Hemsworth et al., 2014). The ethanologenic *E. coli* strain LY01 (Table 1) was equipped with a plasmid carrying an endoglucanase (Cel5A), an exoglucanase (Cel9E), and a β-glucosidase (BGL) from *Clostridium cellulolyticum*. All enzymes were fused with the anchor protein PgsA from *Bacillus subtilis* and could be functionally expressed and localized to the cell surface of *E. coli*. The resulting strain could ferment acid-pretreated corn stover to ethanol with a yield of 95% (Ryu and Karim, 2011).

**Table 1:** **Comparison of ethanologenic *E. coli* strains**.

Strain	Genotype	Ethanol yield (g/g)	Ethanol yield %	Reference
KO3	*pfl*^+^ *pfl*:(*pdc*^+^ *adhB*^+^ Cm^R^)	0.13 g/g glucose	26	Ohta et al. (1991)
KO4	KO3, selected for high Cm^R^	0.56 g/g glucose	>100^a^	Ohta et al. (1991)
KO11	KO4 *frd*	0.54 g/g glucose	>100^a^	Ohta et al. (1991)
KO11	KO4 *frd*	0.46 g/g xylose	89	Yomano et al. (1998)
LY01	KO11, selected for high ethanol tolerance	0.44 g/g glucose	85	Yomano et al. (1998)
LY01	KO11, selected for high ethanol tolerance	0.47 g/g xylose	92	Yomano et al. (1998)
LY 160	KO11, Δ*frd*:*celY*_Ec_ Δ*adhE* Δ*ldhA* Δ*ackA lacA*:*casAB*_Ko_ *rrlE*:(*pdc*_Zm_-*adhA*_Zm_-*adhB*_Zm_-FRT-*rrlE*) *pflB*^+^	0.49 g/g xylose	95	Yomano et al. (2008)
AH003	KO11, Δ*ldhA* Δ*pflA*	0.54 g/g glucose	>100^a^	Hildebrand et al. (2013)
AH003	KO11, Δ*ldhA* Δ*pflA*	0.35 g/g gluconate	98	Hildebrand et al. (2013)
SZ420	Δ*frdBC* Δ*ldhA*, Δ*ackA* Δ*focA*-*pflB* Δ*pdhR*:*pflB*p6-*pflB*rbs-a*ceEF*-*lpd*	0.45 g/g glucose	90	Zhou et al. (2008)
TCS083	Δ*zwf* Δ*ndh* Δ*scfA* Δ*maeB* Δ*poxB* Δ*ldhA* Δ*frdA* Δ*pta*:Kan^−^, carrying plasmid with *pdc_Zm_* and *adhB_Zm_*	0.48 g/g glucose	94	Trinh et al. (2008)
TCS083	Δ*zwf* Δ*ndh* Δ*scfA* Δ*maeB* Δ*poxB* Δ*ldhA* Δ*frdA* Δ*pta*:Kan^−^, carrying plasmid with *pdc_Zm_* and *adhB_Zm_*	0.49 g/g xylose	96	Trinh et al. (2008)
TCS099	Δ*zwf* Δ*ndh* Δ*scfA* Δ*maeB* Δ*poxB* Δ*ldhA* Δ*frdA* Δ*pta* Δ*mdh*:Kan, carrying plasmid with *pdc_Zm_* and *adhB_Zm_*	0.37 g/g glycerol	74	Trinh and Srienc (2009)
TCS099	Δ*zwf* Δ*ndh* Δ*scfA* Δ*maeB* Δ*poxB* Δ*ldhA* Δ*frdA* Δ*pta* Δ*mdh*:Kan, carrying plasmid with *pdc_Zm_* and *adhB_Zm_* evolved with metabolic evolution in glycerol/tetracycline	0.49 g/g glycerol	98	Trinh and Srienc (2009)

*^a^Higher ethanol titers were reached due to ethanol production from the catabolism of complex nutrients. The yield was calculated on initially added sugar*.

Levoglucosan is the most abundant sugar in bio-oil and therefore an attractive fermentation substrate. It can be converted to glucose-6-phosphate by levoglucosan kinase (Kitamura et al., 1991). Hence, Layton et al. (2011) introduced a codon-optimized version of this kinase (LGK) from *Lipomyces starkeyi* YZ-215 into the genome of *E. coli* KO11 (Table 1). The resulting strain could ferment levoglucosan as a sole carbon source to ethanol. Nevertheless, a complete fermentation of the substrate was not achieved. The authors hypothesized that this was due to the high *K*_m_ value (71.2 mM) of LGK or to transport limitations.

In conclusion, *E. coli* was adapted to the utilization of various carbon sources. These are very important improvements of the use of metabolic capacity, and mark essential steps in the development of biotechnological processes with renewable substrates or waste streams as substrates.

## Production of Ethanol

Bioethanol is the single largest biotechnological commodity, which is to the most extend used as a biofuel. Today, it is produced in large scale from glucose or sucrose. A number of microorganisms produce ethanol as a natural fermentation end product, sometimes even in a homoethanologenic type of fermentation (Otero et al., 2007). Ethanol is one of the mixed-acid fermentation end products of *E. coli*. Its production in wild type cells of *E. coli* is catalyzed in a two-step reaction by alcohol dehydrogenase (*adhE*). This enzyme converts acetyl-CoA via acetaldehyde into ethanol and regenerates two NAD^+^ molecules. Ethanol cannot be produced as the sole fermentation end product of *E. coli* wild type, because glucose fermentation leads to two molecules of acetyl-CoA and only two (instead of four) molecules of NADH. Hence, the use of pyruvate formate lyase instead of pyruvate decarboxylase (catalyzing the reaction from pyruvate to acetaldehyde) prohibits *E. coli* from conducting yeast- or *Zymomonas mobilis*-like ethanol fermentation (Knappe et al., 1974; Conway et al., 1987). Therefore, Ingram et al. (1987) transformed a plasmid carrying the pyruvate decarboxylase (*pdc*) and alcohol dehydrogenase genes (*adhB*) from *Z. mobilis* into *E. coli*, which led to strongly increased ethanol production from various sugar compounds (Table 1) (Ingram et al., 1987; Alterthum and Ingram, 1989). Since plasmid-based gene expression demands the addition of antibiotics, Ohta et al. (1991) integrated pyruvate decarboxylase (*pdc*) and alcohol dehydrogenase genes (*adhB*) into the genome of *E. coli* under control of the pyruvate formate lyase promoter (strain KO3). Unfortunately, the resulting lower gene dosage caused decreased ethanol yield, with concomitant higher contaminations by other mixed-acid fermentation end products (Table 1). Therefore, Ohta and colleagues selected first for strains that were *pdc* and *adh* hyperexpressive. This was conducted by using high concentrations of the antibiotic chloramphenicol in the medium, because the chloramphenicol resistance gene (*cat*) was co-introduced with the *pdc* and *adh* gene. This led to strain KO4 in which deletion of the gene for the fumarate reductase *frd* (now strain KO11) resulted in a higher glycolytic flux during xylose fermentation (Tao et al., 2001), lower succinate concentrations, and ethanol yields close to the theoretical maximum (Ohta et al., 1991) (Table 1). Interestingly, strain KO11 was analyzed recently using optical mapping and sequencing. Thereby, Turner et al. (2012) could show that the strain contains approximately 25 tandem repeats of the *pdc*-*adh*-*cat* cassette due to IS10-promoted rearrangements. Nevertheless, a maximum ethanol production yield with glucose and xylose was reached more than 20 years ago. Still, high alcohol concentrations in the fermentation broth were not reached at that point due to ethanol toxicity.

Yomano et al. (1998) isolated and characterized ethanol-tolerant *E. coli* strains carrying pyruvate decarboxylase and alcohol dehydrogenase genes. They inoculated *E. coli* KO11 in liquid LB media and agar plates with rising ethanol concentrations. As a result, an *E. coli* strain (LY01) with an ethanol tolerance of 60 g L^−1^ and 85% ethanol yield could be isolated (parent strain: 35 g L^−1^), which was a benchmark in the evolution of ethanol-producing *E. coli* strains (Yomano et al., 1998). Woodruff et al. (2013) aimed at identifying the genetic factors crucial for enhanced ethanol resistance in *E. coli*. The authors produced plasmid-based genomic *E. coli* libraries with three insert sizes. The libraries were transformed into an ethanol production strain, and it was selected for growth in a medium that was supposed to mimic the conditions in an industrial bioreactor (high ethanol concentration, high osmolarity). Using this method, the authors could identify the three genes *otsA*, *otsB*, and *uspC* as crucial for enhanced ethanol resistance and productivity. In previous studies, UspC was identified as a universal stress protein involved in resistance to osmotic shock conditions as well as to UV-induced DNA damage (Gustavsson et al., 2002; Heermann et al., 2009). The genes *otsA* and *otsB* comprise the trehalose biosynthesis pathway. Trehalose is formed in *E. coli* under various stress conditions, including osmotic stress (Purvis et al., 2005).

Yomano et al. (2008) identified several problems in KO11, limiting the strain’s ability to produce high ethanol amounts in a mineral salt medium. Consequently, strain LY160 was constructed. This strain holds the *Z. mobilis* genes for alcohol production (*pdc*, *adhA*, *adhB*), genes for an endoglucanase from *Erwinia carotovora*, and genes for cellobiose utilization from *Klebsiella oxytoca*. Moreover, Yomano et al. (2008) removed lactate dehydrogenase (*ldhA*), acetate kinase (*ackA*), and the native alcohol dehydrogenase (*adhE*) (Table 1). The resulting strain fermented xylose to ethanol in a minimal salt medium nearly as efficiently as KO11 in an LB medium. An additional deletion of *msgA* (methylglyoxal synthase) enabled a co-fermentation of glucose and xylose (Yomano et al., 2009).

Hildebrand et al. (2013) used KO11 as a starting strain for gluconic acid based ethanol production. Similar to Yomano et al. the authors saw that KO11 contains the genetic repertoire for reactions that could still lead to potential side products. Furthermore, the authors realized that KO11 still contains a functional pyruvate formate lyase and pyruvate dehydrogenase. The latter is strongly downregulated under anoxic conditions but some pyruvate is still converted to acetyl-CoA via this enzyme. Therefore, the authors combined deletions in the corresponding genes for lactate dehydrogenase (*ldhA*), pyruvate formate lyase (*pflA*), and pyruvate dehydrogenase complex (*pdh*) to determine ethanol production with glucose and gluconate as substrate. For both substrates deletion of *ldh* and *pfl* improved the ethanol yield. For gluconic acid, the yield increased from 87.5 to 97.5% while it increased on glucose from 101.5 to 106%. Yields higher than the theoretical maximum were achieved most probably due to the consumption of casamino acids in the LB medium. However, deletion of *pdh* alone or *ldhA* and *pdh* did not have a significant effect on ethanol yield (Hildebrand et al., 2013).

The above-mentioned modifications for ethanol production all included the transgenic expression of *Z. mobilis* genes in *E. coli*. This is why Kim et al. (2007) constructed an *E. coli* K-12 mutant that was not dependent on the expression of foreign genes but retained the ability to produce ethanol as a sole fermentation product. The deletion of the pyruvate-formate lyase and lactate dehydrogenase encoding genes *pflB* and *ldhA*, respectively, inhibited growth without oxygen, but anaerobic growth could be restored after random mutagenesis with ethyl methane sulfonate. In fact, the mutagenesis led to a homoethanologenic strain (Kim et al., 2007). The mutation was later mapped to the pyruvate dehydrogenase gene and most probably enabled expression and activity of this enzyme under anoxic conditions. Zhou et al. (2008) conducted a similar approach. The authors set pyruvate dehydrogenase under control of the native pyruvate formate lyase promoter. Further deletion of the *frd*, *ackA*, *focA*, and *pflB* genes led to strain SZ420, which converts glucose as well as xylose to ethanol with a yield of 90% under anaerobic conditions using only genes and promoters from *E. coli*.

Using elementary mode analysis, Trinh et al. (2008) aimed to broaden the spectrum of growth conditions under which optimal ethanol production with *E. coli* could be obtained. Besides exclusion of routes to other mixed-acid fermentation products, the authors deleted genes encoding glucose-6-phosphate-dehydrogenase (*zwf*), NADH dehydrogenase II (*ndh*), NAD^+^/NADP^+^ dependent malate enzyme (*scfA*, *maeB*), and pyruvate oxidase (*poxB*). The resulting strain (TCS083) was further provided with a plasmid-containing pyruvate dehydrogenase, as well as alcohol dehydrogenase from *Z. mobilis* (pLOI297). TCS083/pLOI297 could then simultaneously convert pentose and hexose sugars to ethanol with a yield of 94–96% (Trinh et al., 2008). Using the same strategy, an *E. coli* strain was engineered to produce ethanol from glycerol aerobically (Trinh and Srienc, 2009). The authors used strain TCS083 with plasmid pLOI297 and further deleted malate dehydrogenase (*mdh*). The evolved strain produced ethanol under low-oxygen conditions with a yield of 0.37 g ethanol/g glycerol consumed, which is close to the theoretical maximum of 0.5 g/g.

In addition to activities achieving ethanol production from glycerol under oxic conditions, Dharmadi et al. (2006) showed that *E. coli* is able to ferment glycerol under certain conditions. Apparently, *E. coli* uses different enzyme sets for the conversion of glycerol to dihydroxyacetone-phosphate under fermentative and respiratory conditions. Without a terminal electron acceptor, the glycerol dehydrogenase (*gldA*) catalyzes, in a NAD^+^-dependent reaction, the formation of dihydroxyacetone, which is subsequently phosphorylated by the activity of the dihydroxyacetone-kinase DHAK (Gonzalez et al., 2008). NAD^+^ as cofactor (instead of a menaquinone-dependent reaction of glycerol-3-phosphate reductase) renders a fermentation from glycerol to ethanol balanced, and ethanol was indeed shown to be the major end product. Glycerol fermentation proceeds best under low pH and in a medium containing low concentrations of phosphate and potassium. Furthermore, the conversion of formate to hydrogen and CO_2_ was revealed as crucial step. The Trchounian group showed that the activity of formate–hydrogen lyase coupled with hydrogenase Hyd-3 is increased at lower pH values. It is possible that lower pH and hydrogenase activity contribute to adjusting a suitable membrane potential under glycerol fermenting conditions (Trchounian et al., 2012; Trchounian and Trchounian, 2013). Recently, it was shown that the rather strict requirements for efficient glycerol fermentation can be bypassed by simply using a co-culture of *E. coli* with the formate consuming methanogen *Methanobacterium formicicum*. The latter organism stabilizes the medium pH and activates glycerol consumption by efficiently removing formate from the medium (Richter and Gescher, 2014).

## Production of Acetate

A homoacetogenic fermentation of sugars is not possible using wild type *E. coli* cells. The ATP-yielding cleavage of the thioester bond in acetyl-CoA does not consume electrons. Hence, NAD^+^ reduced during glucose consumption cannot be regenerated. In other words, a respiratory pathway must be chosen if acetate is supposed to be the sole end product. It is known that acetate accumulates during anaerobic respiration (Trotter et al., 2011), but commercial production favors media without an additional electron acceptor that will accumulate in its reduced form in the fermentation broth. This is why Causey et al. (2003) established a homoacetate pathway in *E. coli* by combining fermentative and respiratory strategies. In a first step, the authors deleted pyruvate formate lyase, fumarate reductase, and lactate dehydrogenase (*focA-pflB*, *frdBC*, *ldhA*) to minimize the formation of fermentation end products. They decreased the production of biomass and ensured ADP recycling via the deletion of parts of the membrane-bound ATPase (Δ*atpFH*). Hence, a soluble cytoplasmic enzyme was engineered that hydrolyzes ATP to ADP. Growth of the resulting strain was dependent on the presence of a fermentable carbon source that could be used for substrate-level phosphorylation. Nevertheless, the quinol pool could be oxidized by oxygen reduction, since the respiratory chain *per se* was still present. The authors modified the strain further by deleting the alcohol dehydrogenase gene *adhE*, which eliminated a competing reaction for the substrate acetyl-CoA. Moreover, the citric acid cycle was blocked via deletion of α-ketoglutarate dehydrogenase. The accompanying auxotrophy for succinate was cured by selecting for spontaneous mutants. The resulting strain produced acetate with a maximum yield of 86% based on glucose consumed (Causey et al., 2003).

One could argue why it is necessary to convert *E. coli* into an efficient acetate producer. Biotechnological production of acetate is based on the oxidation of ethanol by *Acetobacter* strains (Raspor and Goranovic, 2008). This ethanol is mostly produced by yeast. Hence, usage of the genetically engineered *E. coli* strain would first open the process of acetate production to a multitude of carbon sources and would further circumvent a potential competition for the biofuel ethanol. However, the most important point of the study by Causey et al. (2003) is that the authors were able to establish a kind of unbalanced fermentation in *E. coli*. They were able to use the advantages of anoxic fermentative processes (low cell mass, high catabolic rates) and combine them with a respiration-based formation of a product that is more oxidized than the growth substrate. This could be an enabling technology for a variety of other biotechnological production processes that are so far impossible due to insufficient redox balance.

In fact, Causey et al. (2004) used this technology to develop the strain further for pyruvate production. Additional deletion of acetate kinase (*ackA*) and pyruvate oxidase (*poxB*) led to strain TC44. This strain produces 0.75 g pyruvate/g glucose in a mineral salts medium under microoxic conditions. This is the highest yield of pyruvate production in a mineral salt medium reported for *E. coli* so far (Causey et al., 2004).

## Production of Lactate

The production of lactic acid is of biotechnological interest mostly due to the production of polylactic acid- (PLA-) based plastic materials. Since lactic acid bacteria have high nutritional requirements (van Niel and Hahn-Hagerdal, 1999), *E. coli* seems to be better suited for the production of this carboxylic acid.

There are two optical isomers of lactate, and the concentration of each enantiomer determines the properties of the PLA (Wee et al., 2006). Lactic acid can be produced chemically, but only as a mixture of the two isomers. In contrast, microorganisms can build lactic acid in an optically pure d- or l-form (Qin et al., 2012; Wang et al., 2012). With a yield of 0.13 g lactate/g glucose, d-lactic acid is an end product of mixed-acid fermentation in *E. coli* (Clark, 1989; Bunch et al., 1997; Chang et al., 1999). Its formation is catalyzed by the soluble cytoplasmic lactate dehydrogenase *ldhA*, while two membrane-associated forms are responsible for respiratory lactate consumption (Haugaard, 1959; Kline and Mahler, 1965; Tarmy and Kaplan, 1968; Bunch et al., 1997).

Early experiments by Chang et al. (1999) led to a lactic-acid-producing *E. coli* RR1 strain. Competing pathways were blocked by the deletion of phosphotransacetylase (*pta*) and PEP-carboxylase (*ppc*). The double mutant produced lactate with a yield of nearly 90% (0.9 g lactate/g glucose) of the theoretical maximum in a two-phase fermentation process, with an aerobic growth phase and an anaerobic production phase at pH 7. The introduction of l-lactate dehydrogenase from *Lactobacillus casei* on a plasmid caused *E. coli* (Δ*ldhA* Δ*pta*) to produce optically pure l-lactate. With this work, the authors proved it was possible to produce either one of the two optical isomers using *E. coli*. Dien et al. (2001) used a plasmid-based l-lactate dehydrogenase from *Streptococcus bovis* in a pyruvate formate lyase and fermentative lactate dehydrogenase (Δ*pfl* Δ*ldhA*) deficient *E. coli* B derivative. They achieved with this B strain higher lactate yields (93% in contrast to 61–63% in K-12 derivates) and were able to ferment glucose and xylose to l-lactate (Dien et al., 2001).

To prevent the use of plasmids and their need for a constant selective pressure, Zhou et al. (2003) replaced the native *ldhA* gene with the *ldhL* gene from *Pediococcus acidilactici* in the genome of an *E. coli* W3110 mutant. After selection for improved growth, strain SZ85 (Δ*focA-pflB* Δ*frdBC* Δ*adhE* Δ*ackA* Δ*ldhA*) was isolated and showed a lactate yield of 94% with glucose and 82% with xylose as substrate (Zhou et al., 2003). A homoethanologenic *E. coli* B strain (Δ*frdBC* Δ*ldhA*, Δ*ackA* Δ*focA-pflB* Δ*pdhR:pflBp6-pflBrbs-aceEF-lpd*) was reengineered for the production of l-lactic acid from xylose. To prevent the production of ethanol, *adhE* was deleted and *ldhL* from *P. acidilactici* was introduced resulting in strain WL203. After metabolic evolution via growth in an LB-xylose medium, strain WL204 was isolated. This strain produced optically pure l-lactate with a yield of 97% of the theoretical maximum and titers of 62–66 g L^−1^ lactate. However, higher xylose concentrations could not increase the final lactic acid titers, indicating that lactic acid inhibits growth and fermentation of WL204. Moreover, achievable yield (91%) and final titer (42.9 g L^−1^) were significantly lower in a minimal medium compared to a complex medium (Zhao et al., 2013).

In another approach, *E. coli* MG1655 was engineered for efficient, microoxic conversion of glycerol to l-lactic acid through several deletions in the fermentative metabolism. The genes *pta*, *adhE*, and *frd* were deleted to omit formation of side products. The deletion of *mgsA* prevented the generation of d-lactic acid via the methylglyoxal bypass. l-lactate dehydrogenase from *S. bovis* was introduced into the genome of *E. coli*, resulting in an l-lactic acid producer. The authors added copies of the aerobic glycerol consuming pathway (glycerol kinase *glpK*, and glycerol-3-phosphate dehydrogenase *glpD*) on a plasmid and deleted the membrane-bound lactic acid dehydrogenase (*lldD*) to prevent the consumption of l-lactic acid. The resulting strain produces 0.875 g l-lactic acid/g glycerol, which corresponds to a yield of 90% of the theoretical maximum (0.98 g lactic acid/g glycerol) (Mazumdar et al., 2013).

Strain KO11, originally constructed as efficient ethanol producer (Ohta et al., 1991), was later also modified by several deletions (Δ*focA-pflB* Δ*adhE* Δ*ackA*) to enable d-lactate formation (Zhou et al., 2005). Fermentation of glucose and sucrose led to yields of 99% (glucose) and 95% (sucrose) of the theoretical maximum based on metabolized sugar within 144 h. Final lactate concentrations reached up to 1 mol/L, which is comparable to lactic acid bacteria. In this strain (SZ186), no foreign or antibiotic-resistance genes were present, but it lacks the ability to ferment high amounts of sugar completely in a mineral salts medium. Metabolic evolution was used to isolate strain SZ194. This strain was able to convert 12% glucose, which was added to a minimal medium containing 1 mM betaine, completely to d-lactate with a yield of 95%. However, the purity of the d-lactate decreased from 99 to 95% (Zhou et al., 2006). The reason for the impurity was mapped to a methylglyoxal bypass of glycolysis (Grabar et al., 2006). Consequently, deletion of the corresponding gene for the methylglyoxal synthase (*msgA*) abolished the chiral contamination, while the lactate yield remained constant. Interestingly, the base used for pH control is of importance for the final yield. Zhu et al. (2007) were able to increase the final lactate concentration by roughly 17% by using Ca(OH)_2_ instead of NaOH. Using a two-phase production strategy, the authors could reach lactate concentrations of up to 138 g L^−1^ with a strain that was modified by deletion of *aceEF*, *pflB*, *pps*, *poxB*, and *frdABCD*.

## Production of Succinate

Succinate is a citric acid cycle intermediate and a respiration end product if *E. coli* grows anaerobically with fumarate as a terminal electron acceptor (Ingledew and Poole, 1984). Wild type cells produce minor quantities of succinate under fermentative conditions (0.15 g/g glucose) (Kim et al., 2004). The theoretical maximum of succinate production based on the carbon balance is 1.3 g succinate/g glucose (2 mol/mol), since carboxylation of phosphoenolpyruvate (PEP) to oxaloacetate by PEP-carboxylase is a carbon dioxide fixing step. This yield cannot be reached due to the electron and ATP balance of the mixed-acid fermentation pathway (Figure 1). Glucose conversion to two molecules of PEP does not lead to a net production of ATP since two molecules are needed for the activation of glucose to fructose-1,6-bisphosphate, which are produced in the conversion of 1,3-bisphosphoglycerate to 3-phosphoglycerate (Figure 1). Furthermore, the production of two PEP from one molecule of glucose leads to the production of only two instead of four NADH molecules.

Nevertheless, early experiments by Millard et al. (1996) aimed at the overexpression of phosphoenolpyruvate carboxylase and resulted in an increase of the succinate yield to 0.29 g/g glucose. Millard et al. (1996) also suggested that a deletion of the lactate dehydrogenase would further lead to increased succinic acid production. Consequently, Stols and Donnelly (1997) deleted the lactate dehydrogenase and pyruvate formate lyase genes (*ldhA* and *pflB*) with the aim of avoiding synthesis of mixed-acid fermentation side products. This strain grew poorly under anoxic conditions on glucose (Bunch et al., 1997) and showed high intracellular NADH/NAD^+^ ratios (Singh et al., 2009). Nevertheless, growth under anoxic conditions could be restored by overexpression of the NADH-dependent malic enzyme (converting pyruvate to malate) and the availability of CO_2_ (Stols and Donnelly, 1997; Hong and Lee, 2001). The new metabolic route from pyruvate to malate most probably replenished the necessary cofactor NAD^+^ and allowed for ATP production from PEP. Elevated CO_2_ levels did not only support PEP-carboxylase but also malic enzyme activity (Cook, 1983). As a result, succinic acid was produced with a yield of 0.64 g/g glucose. In later experiments, it was observed that the phenotype of the Δ*ldhA* Δ*pfl* strain could also be rescued by a spontaneous mutation (Donnelly et al., 1998). This mutation was later mapped to the glucose transport gene *ptsG* (Escalante et al., 2012). Glucose transport and phosphorylation are possible even in the absence of *ptsG*, since two other systems are encoded in the *E. coli* genome (Figure 1). The reason why a *ptsG* deletion could rescue the phenotype of the *ldhA pfl* deletion mutant is not totally clear yet. One hypothesis is that the rather unspecific import via the other systems results in decreased uptake rates. These would consequently lead to slower formation of glycolysis intermediates and thereby prevent an accumulation of high NADH concentrations before oxaloacetate is reduced to succinate.

Other research groups have tried to increase the production of succinic acid using heterologous expression approaches. Kim et al. (2004) expressed phosphoenolpyruvate carboxykinase (PEP + ADP + P_i_ + CO_2_ → oxaloacetate + ATP) from *Actinobacillus succinogenes* in an *E. coli* phosphoenolpyruvate-carboxylase (Δ*ppc*) deficient strain, thereby succinate production increased roughly twofold (0.26 g/g glucose). Due to the promising results with this enzyme, Singh et al. (2011) used *E. coli* strain AFP111 (Δ*ldhA* Δ*pflB* Δ*ptsG*), deleted *ppc*, and complemented the strain with the above-mentioned enzyme from *A. succinogenes*. As a result, the rate of succinic acid formation as well as biomass production compared to the Δ*ldhA* Δ*pflB* Δ*ptsG* triple mutant could be increased in a dual-phase fermentation by up to 50%, but the succinate yield remained at 0.66 g succinate/g glucose. Singh and colleagues tried to further increase succinate production by deletion of the genes encoding acetate kinase (*ackA*) and phosphotransacetylase (*pta*). However, the resulting mutant did not grow under microoxic or anoxic conditions, which might be due to an accumulation of acetyl-CoA (Singh et al., 2011). Nevertheless, Vemuri et al. (2002) used strain AFP111 and were able to gain succinate yields of 0.96 g succinate/g glucose by combining the overexpression of the *pyc* gene (pyruvate carboxylase from *Rhizobium etli*) in the Δ*ldhA*, Δ*pflB*, and Δ*ptsG* strain with a dual-phase fermentation.

The problem of poor growth as a result of *ackA* and *pta* deletion observed by Singh and colleagues was solved by Sanchez et al. (2005). The authors activated the flow of acetyl-CoA through the glyoxylate pathway by deleting *iclR*, which encodes a transcriptional repressor of genes for the glyoxylate bypass. A further advantage of this second metabolic route is that only one NADH molecule per succinate is necessary, but this comes with the disadvantage of losing one carbon atom in the decarboxylation of pyruvate. Nevertheless, the engineered Δ*ldhA*, Δ*adhE*, and Δ*ack-pta* strain that heterologously expressed the above-mentioned *pyc* gene produced, in an LB medium, succinate with a yield of more than 1 g succinate/g glucose. This strain was recently further improved by coexpression of the formate dehydrogenase from *Candida boindii* (Balzer et al., 2013). This enzyme activity resulted in a decreased production of formate and increased the available pool of NADH. Hence, glucose was consumed faster, fewer side products were detected in the fermentation broth, and succinate was produced from glucose with a yield of 1.1 g/g.

## Conclusion and Outlook

After decades of applied *E. coli* research, a multitude of strains have been developed that allow for the production of mixed-acid fermentation end products with high yields. The crucial and limiting factor regarding the spectrum of producible substances under fermentative conditions is redox balance. Unbalanced fermentations were so far only shown using oxygen as an electron acceptor, but by omitting oxidative phosphorylation using a partial ATPase deletion. Further developments in this field seem to be necessary in order to broaden the spectrum of substances that can be produced under anoxic conditions and thereby raise efficiency via a minimal production of biomass and input of energy. An interesting approach was recently shown by Flynn et al. (2010). They used an anode in a microbial fuel cell as an acceptor for a surplus of electrons. Electrodes are exhaustless electron acceptors. They cannot be depleted. Hence, anodic electron transfer could be a sustainable process to broaden the spectrum of producible substances. Unfortunately, microbial fuel cells demand specific electron transport chains to the cell surface. Nevertheless, cell-permeable electron shuttles, such as neutral red or methylene blue, could also couple the metabolism of other microorganisms to anode reduction.

## Conflict of Interest Statement

The authors declare that the research was conducted in the absence of any commercial or financial relationships that could be construed as a potential conflict of interest.
